# Implementing Remote Collaboration in a Virtual Patient Platform: Usability Study

**DOI:** 10.2196/24306

**Published:** 2022-07-28

**Authors:** Jan Kiesewetter, Inga Hege, Michael Sailer, Elisabeth Bauer, Claudia Schulz, Manfred Platz, Martin Adler

**Affiliations:** 1 Institut für Didaktik und Ausbildungsforschung in der Medizin am Klinikum der Ludwig-Maximilians-Universität München Munich Germany; 2 Medical School Universität Augsburg Augsburg Germany; 3 Chair of Education and Educational Psychology Department of Psychology University of Munich Munich Germany; 4 Instruct gGmbH Munich Germany

**Keywords:** collaborative learning, clinical reasoning, webRTC, collaboration, collaborative, decision making

## Abstract

**Background:**

Learning with virtual patients is highly popular for fostering clinical reasoning in medical education. However, little learning with virtual patients is done collaboratively, despite the potential learning benefits of collaborative versus individual learning.

**Objective:**

This paper describes the implementation of student collaboration in a virtual patient platform. Our aim was to allow pairs of students to communicate remotely with each other during virtual patient learning sessions. We hypothesized that we could provide a collaborative tool that did not impair the usability of the system compared to individual learning and that this would lead to better diagnostic accuracy for the pairs of students.

**Methods:**

Implementing the collaboration tool had five steps: (1) searching for a suitable software library, (2) implementing the application programming interface, (3) performing technical adaptations to ensure high-quality connections for the users, (4) designing and developing the user interface, and (5) testing the usability of the tool in 270 virtual patient sessions. We compared dyad to individual diagnostic accuracy and usability with the 10-item System Usability Scale.

**Results:**

We recruited 137 students who worked on 6 virtual patients. Out of 270 virtual patient sessions per group (45 dyads times 6 virtual patients, and 47 students working individually times 6 virtual patients minus 2 randomly selected deleted sessions) the students made successful diagnoses in 143/270 sessions (53%, SD 26%) when working alone and 192/270 sessions (71%, SD 20%) when collaborating (*P*=.04, η^2^=0.12). A usability questionnaire given to the students who used the collaboration tool showed a usability score of 82.16 (SD 1.31), representing a B+ grade.

**Conclusions:**

The collaboration tool provides a generic approach for collaboration that can be used with most virtual patient systems. The collaboration tool helped students diagnose virtual patients and had good overall usability. More broadly, the collaboration tool will provide an array of new possibilities for researchers and medical educators alike to design courses for collaborative learning with virtual patients.

## Introduction

Learning with virtual patients (VPs) is widely popular in medical education. It is an efficient way to give students the opportunity to learn with real-life clinical scenarios [1-3]. This popularity has led to various e-learning solutions with different conceptual backgrounds [3,4]. Some conceptualizations focus on acquiring clinical knowledge [5-8], while others concentrate on immersing the student in a virtual environment to teach medical communication skills [9-12]. Yet another learning goal is to convey the process of how a patient is diagnosed, known as clinical reasoning, which “includes the application of knowledge to synthesize and prioritize information from various sources and to develop a diagnosis and management plan for a patient” [13]. Facilitating clinical reasoning is a key goal of medical schools, yet one that is difficult to reach. Diverse e-learning innovations have attempted to foster clinical reasoning with varying degrees of success [14-18]. A review found that collaborative features enabling students to communicate within learning environments were still limited in medical education [19]. Compared to the large number of studies investigating individual clinical reasoning, only a few studies have investigated the application of collaborative learning (meaning that two or more people learn together, benefiting from one another's resources and skills) to clinical reasoning [19,20]. We understand collaborative clinical reasoning to be “the process in which two or more health care team members negotiate diagnostic, therapeutic, or prognostic issues of an individual patient resulting in an illness or treatment plan (and to reduce uncertainty)” [19].

One study implemented collaborative learning in biomedical courses via an e-learning environment, showing beneficial effects [21]. Another study investigated the collaborative learning of clinical reasoning in a face-to-face setting and found that pairs of medical students using the same computer made faster and equivalently good diagnoses compared to students learning individually [22]. The so-called ICAP (interactive, constructive, active, and passive) framework from Chi and Wylie [23] might explain these results. Chi and Wylie suggested that student engagement can be distinguished into 4 modes. In collaborative learning, students can interact, which is desirable for deep cognitive processing and learning [23]. VP platforms are sometimes used in face-to-face settings, with groups of students working together on a case, often in problem-based learning settings [24]. However, VP platforms in medical education typically focus on the individual student and not on groups of students working together remotely to learn clinical reasoning [24].

In this paper, we describe the underlying rationale and approach to implementing a collaboration tool in the VP platform Casus (Instruct gGmbH) for learning clinical reasoning. Such a tool, being directly implemented into a VP platform, can help researchers to easily design studies and provide evidence on how to optimize collaborative learning for students. In times such as the COVID-19 pandemic, these tools can also provide learning opportunities that might otherwise be missing.

Our aim was to give pairs of students the ability to communicate remotely with each other (ie, while not in the same room) during VP learning sessions. Simultaneously, we considered that the system should be able to track the learning processes of the students. This would enable medical educators to design collaborative courses and researchers to study collaboration in VP environments. As target users of the system, we had in mind clinical educators in all fields of medicine in which collaboration plays an integral role in everyday work. We hypothesized that using a collaborative tool implemented directly within the VP platform would not impair usability of the system compared to an individual learning setting. Further, we aimed to identify whether learning with the tool led to better diagnostic accuracy for dyads of students compared to individual learners.

## Methods

### Technical Approach

Part of the research project FAMULUS (Fostering Diagnostic Competences in Medical Education and Teacher Training Through Adaptive, Online Case-Simulations), funded by the German Federal Ministry of Education and Research, was to enable students to work together, remotely and synchronously, in medical and teacher education. The tool was designed and developed in several steps, which will be described in the following sections and are summarized in Table 1.

**Table 1:** Stepwise overview of the development process of the tool.

Steps	Descriptions
Step 1: Searching for suitable libraries	Identifying potential collaboration tool libraries
Step 2: Implementing the application programming interface	Defining the educational features needed and implementing (1) video communication, (2) text chat, and (3) screen sharing
Step 3: Making technical adaptations	Installing TURN (traversal using relays around network address translators) and STUN (simple traversal of user datagram protocol through network address translators) to ensure the best potential connections between users despite protective firewalls
Step 4: Designing and developing the user interface	Designing each feature (video communication, text chat, and screen sharing) so it could be turned off and on by the educator; implementing an additional onscreen window providing the collaboration functionality
Step 5: Usability testing and comparing diagnostic accuracy	Comparing the original, individual system with the collaborative tool using 6 virtual patients in a group of 45 dyads (ie, 90 students) and a group of 47 students to test for usability and compare diagnostic accuracy

### Step 1: Searching for Suitable Libraries

After a search of available libraries, we decided to use the SimpleWebRTC (andYet Co) library for implementing the collaboration tool. It provided the basis for setting up a conferencing platform and could be extended to include the required features, such as video communication and screen sharing. An additional text chat function was implemented using the message protocol of the SimpleWebRTC library. We had several reasons for using SimpleWebRTC. At the time we chose it, SimpleWebRTC was an open-source library that was completely scriptable and could be used without additional credentials. This was desirable to enable tight integration into the e-learning platform. The library offered detailed documentation for integration and samples, unlike other open-source solutions. Commercial video conferencing and screen sharing tools, such as Google Hangouts (Alphabet Inc), Zoom (Zoom Video Communications Inc), Skype (Microsoft Inc), and Adobe Connect (Adobe Inc), had disadvantages. For example, Zoom usually requires one account and a dedicated administrator to initiate a call. Consequently, scripting (ie, matching two unique students) a significant number of parallel but independent video conferencing sessions would not have been possible with such tools, even though they might be more robust than WebRTC. As scripting and dynamically creating the dyads was an important part of the study setting, we decided against commercial video conferencing tools. Unfortunately, even SimpleWebRTC has now become a commercial service. Nevertheless, the original library remains open source, is self-maintained, and is regularly tested for cross-browser functionality. Comparable open-source libraries like Jitsi (8x8, Inc) [25] offered no significant advantage compared to SimpleWebRTC, although this might change in the future.

### Step 2: Implementing the Application Programming Interface

To implement SimpleWebRTC independently of Casus, we set it up as an application programming interface (API). This also allowed our approach to be applied to other VP or e-learning platforms. Several features of the conferencing tool could be controlled, enabled, or disabled with the JavaScript API: (1) video and audio channels, (2) text-based chat, and (3) screen sharing. From an educational point of view, each of these features was necessary. First, the audio channel let students collaborate in a direct manner, allowing for communication without additionally transcribing speech to text. Video was helpful for establishing a feeling of proximity, despite the actual distance between the students. Reciprocal, synchronous video-based learning, in which two or more students are directly connected and work together at the same time, has been found to increase learning effectiveness [26]. Second, the text-based chat enabled educators and students to access the messages any time after a learning session and follow up on the collaborative activities. To support this aim, we added two features to the text chat: saving chats as text files and restoring the display of older messages after a browser restart. Third, screen sharing was necessary to allow students to work together in dyads on a shared document. In order to structure the collaboration, we designed the screen sharing with a “main” user, who was able to share his or her screen, and a “secondary” user without this option. This made it possible for educators to choose to give students the same or different sets of information to create a need for collaboration.

### Step 3: Making Technical Adaptations

The implemented educational features (scripting, video and audio channels, text-based chat, and screen sharing) required several technical adaptations. To be able to handle a browser reload triggered by the user or refresh the application when a problem occurred, one component of the API used the conferencing tool to save the current state at regular intervals. SimpleWebRTC includes a signaling service responsible for the exchange of metadata and coordination of the communication between connecting client browsers. Our implementation was based on the node.js signalmaster from andYet. Some adaptations were necessary to improve the monitoring options in order to track students’ logins and logouts and implement the text chat. We have detailed the adaptations in Multimedia Appendix 1.

The adapted signal service, based on signalmaster from andYet, and the additions to SimpleWebRTC used the JavaScript API and were open source. They are available on request from Instruct gGmbH. Detailed documentation for each component is available from the GitHub website [27], as is the documentation for STUN (simple traversal of user datagram protocol through network address translators) and TURN (traversal using relays around network address translators) with the coTurn implementation [28]. A detailed description of how the WebRTC standard manages communication can be found at various websites. A good example is the website of HTML5rocks [29]. The signalmaster from andYet was only minimally extended to provide the text chat functionality and logging for debugging.

Except text chat data, signalmaster does not currently store any data. Text chat data are stored in simple JSON (JavaScript Object Notation) text files with a room naming convention, but can be extended if needed.

### Step 4: Designing and Developing the User Interface

**Figure 1:**
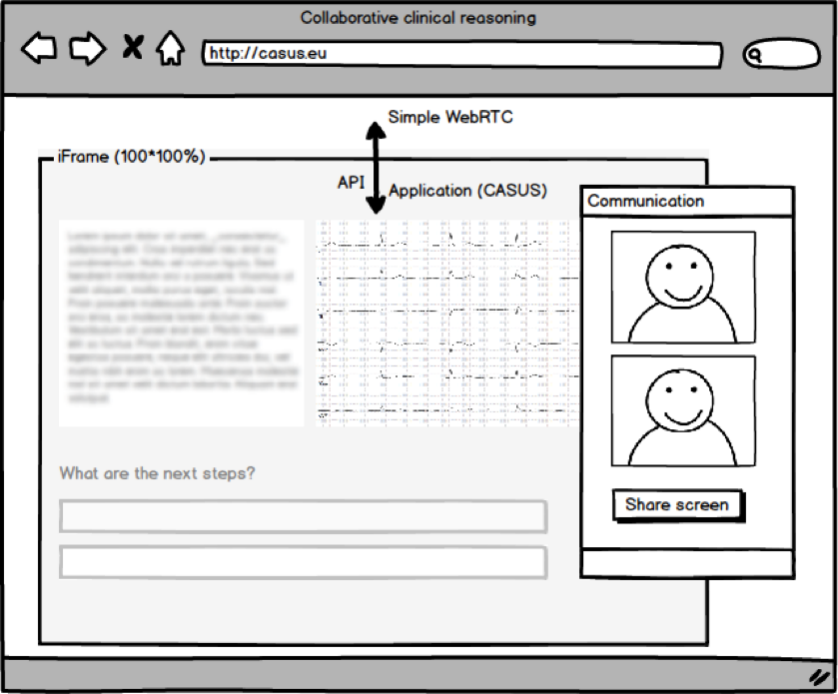
Wireframe model of the integration of Casus with WebRTC. In the foreground two people are communicating; they have the option to share their screens. The application programming interface interacts with SimpleWebRTC. API: application programming interface;

The requirements for the user interface were generated through discussions among the authors to maximize the system’s learning and research possibilities. Students saw the usual Casus user interface with an additional window providing the collaboration functionality (Figure 1 shows a wireframe model of the implementation). Students could use this window to talk to their peers and share their screens. We also implemented a user interface for educators in the Casus course administration area. The setup page enabled educators to set up and configure the collaboration (Textbox 1 shows the available settings).

**Settings available to educators in the user interface.**

- Turn collaboration on or off for a course (for groups of students)
- Define virtual rooms for collaboration (students in the same virtual room can communicate with each other and work on the same virtual patient)
- Enable or disable screen sharing between students (one or multiple students are allowed to share their screens with partners)

To implement the API in Casus, we only needed to implement a few code changes in Java, the main programming language of the server side of Casus. Communication between the host system (Casus in our study) and the communication framework based on SimpleWebRTC is described in detail in Multimedia Appendix 2.

The HTML page for the communication framework contained the host system (again, Casus in our setting) as an iframe. This ensured that even if a user navigated through the host system to different URLs, the communication framework remained open and unchanged, making the communication more stable. Storing the actual URL (eg, in HTML5) in local storage or cookies in order to survive a reload was possible, even though sometimes it could take a few seconds until the participants in the room were reconnected.

With the given API messages, the communication framework could be integrated into any web-based system without needing any internal knowledge of Casus, making it unnecessary to provide details in this paper. Assignment of rooms could be completely controlled and scripted by sending the API these messages: “casuswebrtcopen_*” and “casuswebrtclose_*.”

**Figure 2:**
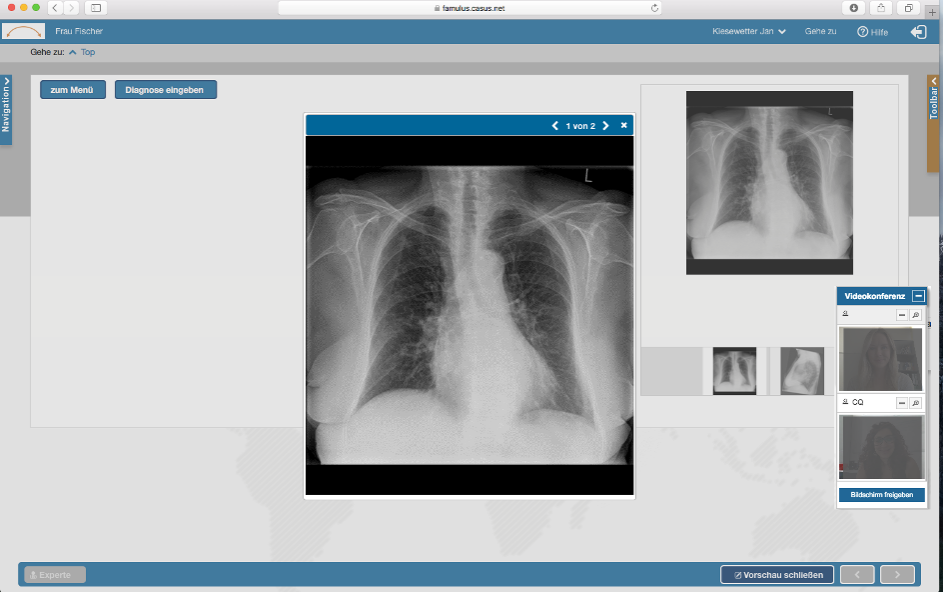
Screenshot of the user interface.

Figure 1 shows the technical implementation, including all components. The user interface is depicted in Figure 2.

### Step 5: Usability Testing and Comparing Diagnostic Accuracy

In two cohorts, we implemented the collaboration tool for students with six VPs. In the first cohort, 45 dyads diagnosed the cases together, while in the second cohort, 47 individual students diagnosed the same VPs on their own (Table 2 shows details of the two cohorts’ demographic data). We invited all medical students at the Ludwig-Maximilian University of Munich between their third and fifth year to participate via email. Allocation to dyad or individual learning was randomized according to the booking date of the participants. Overall, 270 VP sessions per group (45 dyads [ie, 90 students] times 6 VPs, and 47 students working individually times 6 VPs minus 2 randomly selected deleted sessions) were included in the analysis. Participation was voluntary and anonymous. Students received €35 (US $36.54) for approximately 3 hours of their time.

**Table 2:** Descriptive statistics of the two cohorts included in the sample. All participants were between their third and fifth year of medical school.

Cohort	Number of participants, N	Mean age, years	Female, n (%)
First cohort: dyads	90 (ie, 45 dyads)	25	63 (70)
Second cohort: individual learners	47	24	33 (71)

The students in the dyads did not know each other before the study. They were allowed to exchange names but not year of medical school. The VPs were based on texts and images of clinical scenarios, with no video or audio content. We did not allow for text chat in this study, as we wanted the focus of the investigation to be on screen sharing, video, and audio collaboration. Dyads and individuals worked with the same VPs. Three of the VPs exhibited the leading symptoms of back pain (these were all male), while the other 3 exhibited the leading symptoms of fever (these included 1 male and 2 females). All cases were of medium difficulty (mean difficulty range 0.45 to 0.69; these are standard units, defined as percentage correct), as previously tested with individual students, and included all necessary visual content (eg, x-rays and computerized axial tomography scans), although no pictures of the VPs were provided. Dyads of 2 students were connected via the collaboration tool, with each student being the main user for 3 VPs and the secondary user for the other 3 VPs, meaning that students had to balance their teamwork effort. Within the dyads, students had to settle on one final diagnosis for each VP. After reading the patient information, the teams of 2 had to choose which of the 23 available tests they wanted to look at next. They could proceed with as many tests as they wished before making a diagnosis.

### Ethical Approval

Ethics approval and consent to participate were granted by the Ethics Committee of the Medical Faculty of Ludwig-Maximilian University of Munich (study 17-250). All participants gave written consent to participate in the study. We have obtained written consent by the persons identifiable in Figure 2 to publish their images.

### Data Analysis

To evaluate the usability of the collaboration tool and its integration into the Casus VP platform, we used the System Usability Scale (SUS). The SUS is a reliable 10-item measure of usability, scored from 1 to 5, which in total can be extrapolated to grades from 10 to 100 [30]. The web-based questionnaire was implemented in Casus.

We analyzed the data of 137 students who completed the SUS [30] and compared the 45 dyads with the 47 individual learners. We compared usability and diagnostic accuracy using SPSS with two ANOVAs; alpha-error level was set at *P*=.05.

## Results

### Usability Testing

We hypothesized that using the collaboration tool, which is implemented directly within the VP platform, would not impair usability of the system compared to an individual learning setting. The descriptive results of the original SUS scores (scored from 1-5) are presented in Table 3. As recommended by the SUS developers, we transformed the original SUS scores to percentage scores, meaning that high scores always signify good usability. There was no significant difference between the students working individually (mean score 81.28, SD 1.01), and the students working in dyads (mean score 82.51, SD 1.56). For both dyad and individual learners, the SUS score averaged a letter grade of B+ (ie, acceptable). We encountered no major technical issues during the study sessions. The students in dyads collaborated in all sessions and used the screen sharing option in both directions (ie, as both main users and secondary users). The results support the assumption that the implemented collaboration tool would not impair usability of the system compared to individual learning.

**Table 3:** Descriptive statistics of the System Usability Scale (N=137 respondents).

System Usability Scale item	Mean score (SD)
I think that I would like to use this system frequently.	3.72 (1.01)
I found the system unnecessarily complex.	1.93 (0.85)
I thought the system was easy to use.	4.03 (0.98)
I think that I would need the support of a technical person to be able to use this system.	1.36 (0.79)
I found the various functions in this system were well integrated.	3.74 (0.94)
I thought there was too much inconsistency in this system.	2.10 (0.93)
I would imagine that most people would learn to use this system very quickly.	4.28 (0.85)
I found the system very cumbersome to use.	1.88 (1.09)
I felt very confident using the system.	4.12 (0.90)
I needed to learn a lot of things before I could get going with this system.	1.54 (0.87)

### Diagnostic Accuracy Comparison

We investigated whether learning with the collaboration tool led to better diagnostic accuracy for dyads of students compared to individual learners. Of 270 VP sessions per group (45 dyads [ie, 90 students] times 6 VPs, and 47 students working individually times 6 VPs minus 2 randomly selected deleted sessions), students made successful diagnoses in 143 cases (53%, SD 26%) when working individually and 192 (71%, SD 20%) when working in dyads. The dyads working with the collaboration tool achieved significantly higher diagnostic accuracy compared to the individual learners (*P*=.04, η^2^=0.12).

## Discussion

### Principal Findings

We have successfully implemented a tool for remote collaboration into a VP platform, enabling students to learn together. We implemented VPs that enabled remote synchronous collaborative learning of clinical reasoning in the Casus VP system. The development of a generic API allowed the collaboration tool to be used with other e-learning platforms or learning management systems. The results of the usability questionnaire show that there was no significant usability impairment when working with the tool. Subjectively, the usability was even slightly higher. Usability in our study was comparable to that in usability tests routinely performed with the Casus system for individual learners [31,32]. This provides initial evidence that the additional technical aspects of the collaboration tool did not decrease the usability of Casus. The Casus tool was designed some years ago for use by individual students [33,34]. With the present effort to expand the use of Casus to include collaborative learning, we provide initial evidence that working in dyads increases the diagnostic accuracy of students. This could indicate that students working in dyads engaged more in the “interactive learning mode” defined in Chi’s ICAP framework [22]. However, more in-depth research is needed to provide more evidence.

### Comparison With Prior Work

We are aware that other tools for collaboration are available, including commercial online platforms and open-source platforms such as Jitsi. All of these platforms work well on their own, but have several disadvantages that limit their use in educational settings. First, the platforms require login and user identification in a separate browser window from the VP platform. These tools work well when collaboration itself is the goal, but the VP environment provides detailed information on the patient and asks users to respond to questions and provide a diagnosis. An additional browser window complicates an already complex user interface and erects a barrier for educators seeking to incorporate collaborative clinical reasoning into their courses. Second, educators cannot monitor students’ collaboration using the commercially available platforms. For example, there is no way of knowing whether the students are actually connected through a third-party platform. Third, educators do not receive any data regarding collaborative learning, which limits research when utilizing these tools.

For assessment purposes, VPs have proven more effective than standardized patients [19]. For learning purposes, a systematic review found that VPs are advantageous for learning skills, especially clinical reasoning, and comparably effective for learning knowledge [2]. For collaborative clinical reasoning, future research is still needed. For example, it remains to be determined whether and how chat-based communication can be used and how it influences collaboration. In smaller courses, however, this might distract from the task at hand. From an educational perspective, the amount of information each learner receives also needs to be explored. Users should have sufficient information for collaboration, but not be overwhelmed by the amount of information [33,34].

### Limitations

We are aware that our tool has some limitations. Thus far, no courses can be guided as a whole; every user needs to be configured separately. Additionally, currently only the educator, not the learner, can determine the roles of main and secondary user and the amount of information each user is provided with. This study included students in their third to fifth years of medical school because the VPs were designed for these years. Thus, we do not yet know whether our results are transferrable to earlier or later training, or to postgraduate training.

### Conclusions

Our collaboration tool was specifically developed to support collaborative clinical reasoning education with VPs. However, the tool’s design also allows for other simultaneous collaboration scenarios, including in nonmedical domains. For example, the tool could be applicable in teacher education, with two teacher education students having to determine a virtual child’s reading proficiency. The tool enables video-supported communication with optional screen sharing between students and allows educators to easily activate or deactivate the collaboration feature. It also runs on all major internet browsers without any installation procedure.

Our collaboration tool helps students work together to apply content knowledge through training with VPs. The tool provides the necessary basis for using learning analytics to track students’ knowledge progress and collaborative clinical reasoning skills. As a future step, we could use the tool and API to guide students through a VP curriculum designed to impart both knowledge aspects. More broadly, the tool provides new possibilities for researchers and educators alike for designing courses, sharing homework assignments, and researching questions for collaborative learning.

## Appendix

Multimedia Appendix 1 Adaptions to SimpleWebRTC signalmaster from andYet and the additions to SimpleWebRTC.

Multimedia Appendix 2 The communication between the host system (in our studies CASUS) and the communication framework based on SimpleWebRTC with standard JavaScript.

## Abbreviations

API: application programming interface
FAMULUS: Fostering Diagnostic Competences in Medical Education and Teacher Training Through Adaptive, Online Case-Simulations
ICAP: interactive, constructive, active, and passive
STUN: simple traversal of user datagram protocol through network address translators
SUS: System Usability Scale
TURN: traversal using relays around network address translators
VP: virtual patient

## Data Availability

The data sets generated and analyzed during the current study are available from the corresponding author on reasonable request.
