# Supplementary material for: Implementing Remote Collaboration in a Virtual Patient Platform: Usability Study
Source: JMIR Med Educ. 2022 Jul 28;8(3):e24306. doi: 10.2196/24306 (PMC9377431; doi:10.2196/24306)
Supplement: Multimedia Appendix 1 [file mededu_v8i3e24306_app1.docx]

Multimedia Appendix 1: Adaptions to SimpleWebRTC signalmaster from andYet and the additions to SimpleWebRTC

SimpleWebRTC usually tries first to connect to the client browser directly (peer-to-peer) through the signaling service. However, under certain circumstances, the connection can become complex due to routers, proxies, and firewalls between participating clients. WebRTC [20] provides two options for such scenarios: STUN (Simple Traversal of User datagram protocol through Network address translators) handles more complex communication coordination, although the participating clients continue to communicate directly peer-to-peer. The peer-to-peer communication may, however, not work with more protective firewalls. In such cases, TURN (Traversal Using Relays around Network address translators) is necessary, which means that all traffic goes through an additional central relay service. A TURN server needs substantial bandwidth and power, because all communication channels (video, audio, screensharing) will go through this service. We installed both TURN and STUN servers using the so-called CoTurn implementation on a virtual server without any adaptations. This approach has an advantage for the learner: If none of the learners have installed an extensive protective firewall, the learners are directly connected and their individual bandwidth forms the limit of their connection. If, however, one or more of the learners are working behind a more protective firewall, the additional server is utilized, but a connection is still possible.
