# Supplementary material for: Implementing Remote Collaboration in a Virtual Patient Platform: Usability Study
Source: JMIR Med Educ. 2022 Jul 28;8(3):e24306. doi: 10.2196/24306 (PMC9377431; doi:10.2196/24306)
Supplement: Multimedia Appendix 2 [file mededu_v8i3e24306_app2.docx]

Multimedia Appendix 2: The communication between the host system (in our studies CASUS) and the communication framework based on SimpleWebRTC with standard JavaScript

The communication between the host system (in our studies CASUS) and the communication framework based on SimpleWebRTC with standard JavaScript

uses the following methods:

- window.addEventListener("message", *listener* );
- top.postMessage(*message*, "*");

*listener* := JavaScript function within the communication framework

*message* := string

Following messages can be sent by the LMS to the communication framework and define the API:

- Prefix “casuswebrtcopen_" with Parameters
  - actual timestamp
  - requested communication room name,
  - + optionally nickname, language, and flags for enabling/ disabling feature of communication (screensharing, audio, video, text chat)

This event opens the communication channel with room name provided in the request. By this the LMS can decide about which user is added to which communication channel/room.

- Prefix “casuswebrtclose_“ for closing the active communication channel/room
- Prefix “casusurl_“ with Parameters:
  - actual timestamp
  - URL within CASUS:

This enables the communication framework to safe the status and store the actual URL and restore it when a browser reload is performed by the user

- „casuswebrtc_disablescreensharing“ and „ casuswebrtc enablewebrtcscreensharing“ are extra messages to enable / disable screensharing functionality dynamically when needed.
